# Relations between short-term memory and the within-subject variability of experimental pain intensity reports: Results from healthy and Fibromyalgia patients

**DOI:** 10.1371/journal.pone.0277402

**Published:** 2022-11-16

**Authors:** Rita Canaipa, Amira Khallouf, Ana Rita Magalhães, Rafael Teodoro, Vanessa Pão-Mole, Mariana Agostinho, Fernando Pimentel-Santos, Liat Honigman, Roi Treister

**Affiliations:** 1 Universidade Católica Portuguesa, CIIS, Center for Interdisciplinary Health Research, Institute of Health Sciences, Catholic University of Portugal, Palma de Cima, Lisbon, Portugal; 2 Universidade Católica Portuguesa, Institute of Health Sciences, Catholic University of Portugal, Palma de Cima, Lisbon, Portugal; 3 CEDOC, NOVA Medical School, Universidade Nova de Lisboa, Department of Rheumatology, CHLO, Hospital Egas Moniz, Lisbon, Portugal; 4 The Clinical Pain Innovation Lab, The Cheryl Spencer Department of Nursing, Faculty of Social Welfare and Health Sciences, University of Haifa, Haifa, Israel; University of Catanzaro, ITALY

## Abstract

While factors contributing to between-subjects differences in pain have been studied extensively, factors contributing to the within-subjects variability of pain reports are yet unexplored. The aim of this investigation was to assess possible associations between short-term memory and the within-subjects variability of pain reports in healthy and chronic pain patients. Healthy participants were recruited at the University of Haifa, Israel, and Fibromyalgia patients were recruited at a rheumatology department in a central hospital in Lisbon, Portugal. Following consent, both cohorts underwent the same procedures, including the digit-span test, assessing short-term memory, and the FAST procedure, assessing within-subject variability of pain intensity reports in response to experimental pain. One-hundred twenty-one healthy volunteers and 29 Fibromyalgia patients completed the study. While a significant correlation was found between the within-subjects variability and the total score of the short-term memory task (Spearman’s *r* = 0.394, *P* = 0.046) in the Fibromyalgia group, a marginal correlation emerged in the healthy cohort (*r* = 0.174, *P* = 0.056). A possible interpretation of these results is that in the patients’ group, at least some of the within-subjects variability of pain intensity reports might be due to error measurement derived by poorer short-term memory, rather than true fluctuations in perception.

## Introduction

Pain arises from a complex interplay of ascending sensory signals that are processed and heavily modulated by the peripheral and central nervous system. Not surprisingly, this process results in huge between-subjects variability. This variability is well recognized, and much research has focused on identifying factors contributing to between-subject variability of various pain characteristics [1–3]. It is well recognized that physiological, psychological, social, and genetic factors play a role in this between-subject variability [4–8].

Pain varies not only between individuals but also within an individual. This within-subject variability is not surprising: clinical pain does fluctuate from day to day or even within a day, from morning to evening or even from hour to hour. The same is true when an experimental noxious stimulus is applied [9–11]. Subjects will show some variability in their responses to the same stimulus repeated over time. While such within-subjects’ fluctuations could be derived from real changes in perception due to endogenous modulation processes, it may be that at least some of this variability is due to the cognitive process of assessment and reporting of pain [12–14]. Unlike between-subject variability, which has been extensively studied, there is virtually no information about cognitive factors that contribute to the tendency of reporting fluctuating pain, either in clinical settings or in response to experimental stimuli.

As far as we know, the first to highlight the potential clinical relevance of the tendency to report fluctuating intensities of clinical pain were Harris and colleagues [15]. They retrospectively analyzed data from a study in which the effect of milnacipran on pain in a group of fibromyalgia patients was assessed. These researchers focused on the day-to day variability of pain intensity reports documented by the patients via a pain diary at baseline, before they enrolled into the treatment phase. For each subject, the standard deviation of the pain reports collected during the two baseline weeks was calculated. They found that the within-subject variability was distributed normally, with most of the participants demonstrating moderate variability and with fewer participants at either extreme (i.e., expressing low or high variability). They also found that the within-subject variability at baseline was correlated with the response to placebo but not with the response to the treatment [15]. Harris’s first finding was later replicated by Farrar et al. [16], who conducted a meta-analysis of 12 clinical trials on post-herpetic neuralgia (*n* = 1514) and painful diabetic peripheral neuropathy (*n* = 1226). They found that the greater the within-subject variability in the 7-day baseline pain report, the larger the placebo response. However, a recent, smaller meta-analysis of 3 studies with total n of 160 neuropathic pain patients could not replicate this association [17], while another report, based on 139 chronic low-back patients from two studies found very weak associations [18]. The attention given to within-subjects fluctuations in clinical pain intensity is part of a growing interest in the collection of subjects’ reports of their own behavior and experiences at multiple moments in real time and in the participants’ natural environment (rather than in a clinic or lab). This research approach, termed *ecological momentary assessment*, has been investigated mostly in the psychological domain [19, 20] and, more recently, in the pain arena [21–24].

Another approach to study the within-subject variability of pain reports could be based on experimental pain procedures. The focused analgesia selection test (FAST) paradigm measures the within-subject pain intensity variability in response to thermal stimuli of multiple different intensities, applied repeatedly [25]. The random application of those stimuli allows calculating the within-subject variability of pain reports of each individual. In a recent study [26], we found that the day-to-day variability of clinical pain reports and the variability captured in the FAST paradigm were correlated with each other, and both predicted the placebo response.

Given that the magnitude of within-subject variability is derived from changes between current pain and previously reported pain (last day, in case of clinical pain, or few seconds ago, in case of the FAST procedure), it is reasonable to assume that memory might be a factor contributing to the fluctuations in reported pain. The magnitude of within-subject variability in response to experimental pain is affected by the differences between reports of current pain which are mentally compared with recently experienced pain [27]. Hence, memory might reasonably be related to the fluctuations in reported pain. The current report summarizes the results of 2 studies, one in a healthy population and the other in Fibromyalgia patients. The rational was to investigate whether possible relations between short-term memory and the within-subject variability of pain intensity reports in response to experimental pain exist across different cohorts.

## Methods

This report includes data from two observational studies conducted by the two collaborative groups located in Haifa, Israel and Lisbon, Portugal. Both groups designed and conducted the studies in collaboration and applied the same methods. The Haifa cohort consisted of healthy participants, and the Lisbon cohort comprised with Fibromyalgia patients. These two laboratories work in collaboration in the last six years, and principal investigators and students were mutually trained on a regular basis to ensure consistency across sites.

### Subjects

#### Healthy participants

Approval for conducting the healthy volunteers’ study was given by the university of Haifa ethical committee. Participants, students from the university who met the following criteria were invited to participate: 1) literate adults aged above 18; 2) absence of acute pain at the time of evaluation and any chronic pain condition; 3) no reports of psychiatric, cognitive, and/or neurological disorders; 4) no history of alcohol or drug abuse/dependence; 5) no use of analgesics in the last 48 hours or use of regular consumption of any medication, except for oral contraceptives. Participants provided written informed consent at the beginning of the study.

#### Fibromyalgia patients

The study was approved by the Ethical Committee of the Centro Hospitalar de Lisboa Ocidental, Hospital de Egas Moniz, EPE. Patients diagnosed by rheumatologists according to the 1990 American College of Rheumatology [28] classification criteria and the Wolfe et al. [29] diagnostic criteria were invited to participate. Inclusion criteria included: no change in medication consumption four weeks prior to enrollment into the study, over 18 years of age, female, and capable of providing written informed consent. Exclusion criteria included current pregnancy or breastfeeding, any persistent or severe infection within 30 days of baseline, formal diagnosis of psychiatric conditions, history of rheumatic disease beyond Fibromyalgia, any uncontrolled medical condition, and history or signs of demyelinating disease.

### Instruments and procedures

#### Demographic information

All participants from both cohorts provided demographic information via a short survey which included the following information: age, gender, years of education, and marital status.

#### Assessment of within-subject variability of pain intensity reports

The FAST procedure assesses the within-subject variability of pain intensity reports. Thermal noxious stimuli of varying intensities were randomly applied to the ventral surface of the subject’s nondominant arm. The Medoc Thermal Sensory Analyzer II using a Peltier element-based thermode (30 × 30 mm) was used to induce thermal noxious stimuli. The subjects rated the pain intensity in response to each stimulus on a 0–100 numerical rating scale (NRS), where 0 = “no pain” and 100 = “the worst pain imaginable.”. Before beginning with the FAST procedure, to assure that participants fully understood the task, a familiarization session was conducted, during which participants were exposed to 3 stimuli representing low, medium and high intensity, allowing them to practice and ask clarification questions, if needed.

During the FAST procedure, the temperature raised from a baseline of 32°C to a peak temperature lasting for 3 seconds at 1 out of 7 temperatures (44, 45, 46, 47, 48, 49, or 50°C). Based on vast experience with the FAST procedure, there are no concerns for skin burns due to the short stimuli durations. To improve blinding, we had a slightly different rate of temperature ramping change (both up and down) in each temperature, resulting in an entire stimulus duration of 8 seconds, for all temperatures. After each stimulus, the subject was asked to report the pain intensity perceived. Each temperature was presented 7 times in a random block-ordered design (total of 49 stimuli), according to a previously described protocol [25]. To minimize sensitization and/or habituation effects, the intervals between stimuli are relativity long (20 seconds) and the location of the thermode is slightly changed every 10 stimuli.

The Pearson’s coefficient R^2^ value and the intraclass correlation coefficient (ICC) were calculated as the FAST outcomes. R^2^ is based on power regression, measuring the agreement (or correspondence) between actual and predicted scores. The ICC measures the agreement or consistency in responses to the same stimulus over several presentations independently of their order. Higher ICC and *R*^2^ scores represent lower within-subject’s variability [25].

### Short-term memory assessment via the digit span

The Digit Span Task, a subtest of the Wechsler Memory Scale, is designed to assess short-term memory [30]. It is composed of two tasks: the direct (or forward) test and the reverse (or backward) tests. The forward-order version measures immediate verbal memory, whereas the reverse-order test is one of the simplest and most used tests to assess working memory. Working memory is a type of short-term memory that requires manipulation of information.

In the forward-order task, the participant is asked to repeat, in the same order, strings of digits of increasing length, said by the examiner. The first level is a list of 2 numbers, and the last level a list of 9 numbers. The participants get 2 attempts for each level; they are allowed a second attempt only if they fail to correctly repeat the sequence on the first try. The test ends when the participant fails the sequence of digits in the 2 attempts at the same level or if the person successfully reproduces the 9-number string. In the second task, reverse order, the participant is asked to repeat in reverse order strings of digits of increasing length, said by the examiner. The criteria for ending this test are the same as the forward-order test. The score of each task is the number of digits in the string that the participant was able to complete. A total score combines the scores of the 2 tasks.

### Additional information collected only from the fibromyalgia patients

#### Clinical information

Clinical information from Fibromyalgia patients was collected to characterize the patients’ health status. All Fibromyalgia patients reported information on symptom duration and medication consumption. Participants’ medication regimen was organized according to four categories: *analgesics* (nonsteroidal anti-inflammatory drugs, weak opioids or other analgesics); *psychotropic* (anticonvulsants, antidepressants, anxiolytics, antipsychotics, amphetamines); *rheumatic* (antirheumatics, biological, corticosteroids); and *hormonal* (thyroid-related, oral contraceptives, menopause-related).

The following clinical questionnaires were used to characterize the clinical cohort:

#### Brief Pain Inventory (BPI)

The BPI is a self-report measure assessing pain from a multidimensional perspective [31]. It includes 15 items assessing the existence of pain, its severity, its location, the therapeutics used, and the functional impact of pain. The BPI has two subscales: *severity*, which measures the intensity of pain, and *interference*, measuring the functional impact of pain on several daily life activities. The subject answers each question on an 11-point scale, ranging from 0 = “no pain” to 10 = “pain as bad as one can imagine.” Higher scores indicate higher severity and interference of pain. In this population, we used the Portuguese version of the BPI, which has demonstrated good psychometric properties [32].

#### Fibromyalgia Impact Questionnaire (FIQ)

This questionnaire is used to assess the health problems related to Fibromyalgia and its impact on daily living [33]. It includes information about function, overall impact, and symptoms. The FIQ physical functioning domain is based on the patient’s answers to 11 items, rated on a 4-point scale ranging from “Always” to “Never.” The overall impact is calculated from two items that ask about the number of days in the last week during which the patient felt good and was able to work. The symptoms domain of the FIQ measures the presence of 10 symptoms on a 10 cm visual analog scale. Accordingly, the maximum score of the FIQ is 100. Higher scores indicate a higher burden of Fibromyalgia in the patient’s life. The Portuguese version, which was developed by Rosado et al. [34] and demonstrated good psychometric properties, was used in this study.

#### 36-Item Short Form Health Survey (SF-36)

The SF-36 measures the participant’s perception of general health [35]. It consists of 36 items measuring 8 health domains: physical function, limitations related to physical health problems, bodily pain, general health, vitality, social functioning, limitations related to emotional problems, and emotional well-being. Two subscales can be obtained from these scores: a physical component summary and a mental component summary. Scores for both subscales can range from 0 to 100. The higher the score, the better the health and quality of life perceived by the patient. The Portuguese version of the SF-36, which has shown good psychometric properties [36], was used in this study.

#### Hospital Depression and Anxiety Scale (HADS)

This tool assesses anxiety and depression in physically ill populations [37]. HADS has 14 items answered on a 4-point scale, from 0 to 3. Two subscales can be obtained from HADS: depression (7 items) and anxiety (7 items). Total scores for each of these subscales can range from 0 to 21. A higher score indicates higher depression and/or anxiety. The validated Portuguese version of this instrument was considered adequate [38] and was used in the patient cohort.

### Statistical analyses

Analyses were conducted using SPSS for Windows version 25 (IBM Corp., Armonk, NY, 2020). Descriptive statistics were used to summarize the demographic information. The 2 groups’ demographics were compared using independent *t*-tests or chi-square tests, as applicable. Visual inspection of distribution curves, followed by Kolmogorov-Smirnov and Shapiro-Wilk tests, showed that both pain variability and short-term memory outcomes were not normally distributed. Hence, we conducted nonparametric tests to compare those outcomes between the groups and their correlation. Friedman’s tests (followed by Wilcoxon post hoc test, when applicable) were used to analyze the differences in pain intensity measures reported during the FAST procedure. Mann-Whitney U tests were used to compare pain sensitivity, pain variability, and short-term memory between groups. Spearman’s correlation analyses were used to assess the relationship between the within-subject variability and working memory. *P*-values of ≤ 0.05 were regarded as significant.

## Results

### Participant characteristics

Among the 121 healthy individuals, 74 (61.2%) were women (Table 1). The mean age was 24.13 ± 3.37 years, ranging between 20 and 45 years old. They had a mean of 14.89 ± 1.66 years of education, and most group members were not married (82.6%).

**Table 1:** Demographic data of the healthy cohort (HC) (n = 122) and Fibromyalgia (FM) (n = 29) cohorts.

Characteristics	Healthy Controls	Fibromyalgia	p-value
**Age**			<0.001
Mean±SD	24.13±3.37	50.41±10.34	
Range	20–45	30–76	
**Years of Education**			<0.001
Mean±SD	14.89±1.66	10.75±4.91	
Range	12–20	4–19	
**Gender**			<0.001
Female	74 (61.16%)	29 (100%)	
Male	47 (38.84%)	0 (0%)	
**Marital Status (%)**			<0.001
Married	16 (13.22%)	19 (65.5%)	
Other	105 (86.78%)	10 (34.5%)	

Thirty women diagnosed with Fibromyalgia were recruited. From this sample, 1 subject was excluded because of personal schedule constraints. Thus, the final cohort consisted of 29 fibromyalgia patients, with a mean age of 50.41 ± 10.34, ranging between 30 and 76 years old. They had a mean of 10.75 ± 4.91 years of education. The majority of patients were married (65.5%).

### Patient characteristics

The mean disease duration (since symptoms have emerged) of the fibromyalgia patients was 13.96 ± 11.21 years (range 2–46 years). Details of the 6 clinical questionnaires and the medication used can be found in Table 2. Most of the patients used analgesics (79.3%), and slightly more than half were using psychotropics (51.7%).

**Table 2:** Clinical characteristics of fibromyalgia patients (n = 29).

Characteristics	Score
**Years of symptoms: mean±SD**	13.96±11.21
**Medication**: number of patients taking medication (%)	
Analgesic	23 (79.3%)
Psychotropic	15 (51.7%)
Rheumatic	3 (10.3%)
Hormonal	3 (10.3%)
**BPI: mean±SD**	
Severity Score	5.51±1.99
Interference Score	5.81±2.26
**FIQ: mean total score ± SD**	64.29±17.32
**HADS: mean±SD**	
Anxiety Score	11.46±3.86
Depression Score	8.5±3.88
HADS Total Score	19.96±7.11
**SF-36: mean ± SD**	
Physical Component Score	30.81±22.55
Mental Component Score	42.92±24.23
SF-36 Total Score	36.87±22.29

Medication: *analgesic* (nonsteroidal anti-inflammatory drugs, weak opioids, other analgesics); *psychotropic* (anticonvulsants, antidepressants, anxiolytics, antipsychotics, amphetamines); *rheumatic* (antirheumatics, biological, corticosteroids); *hormonal* (thyroid-related, oral contraceptives, menopause-related); SD = standard deviation.

### Pain sensitivity and within-subject pain variability in response to experimental pain via the FAST procedure

**Fig 1:**
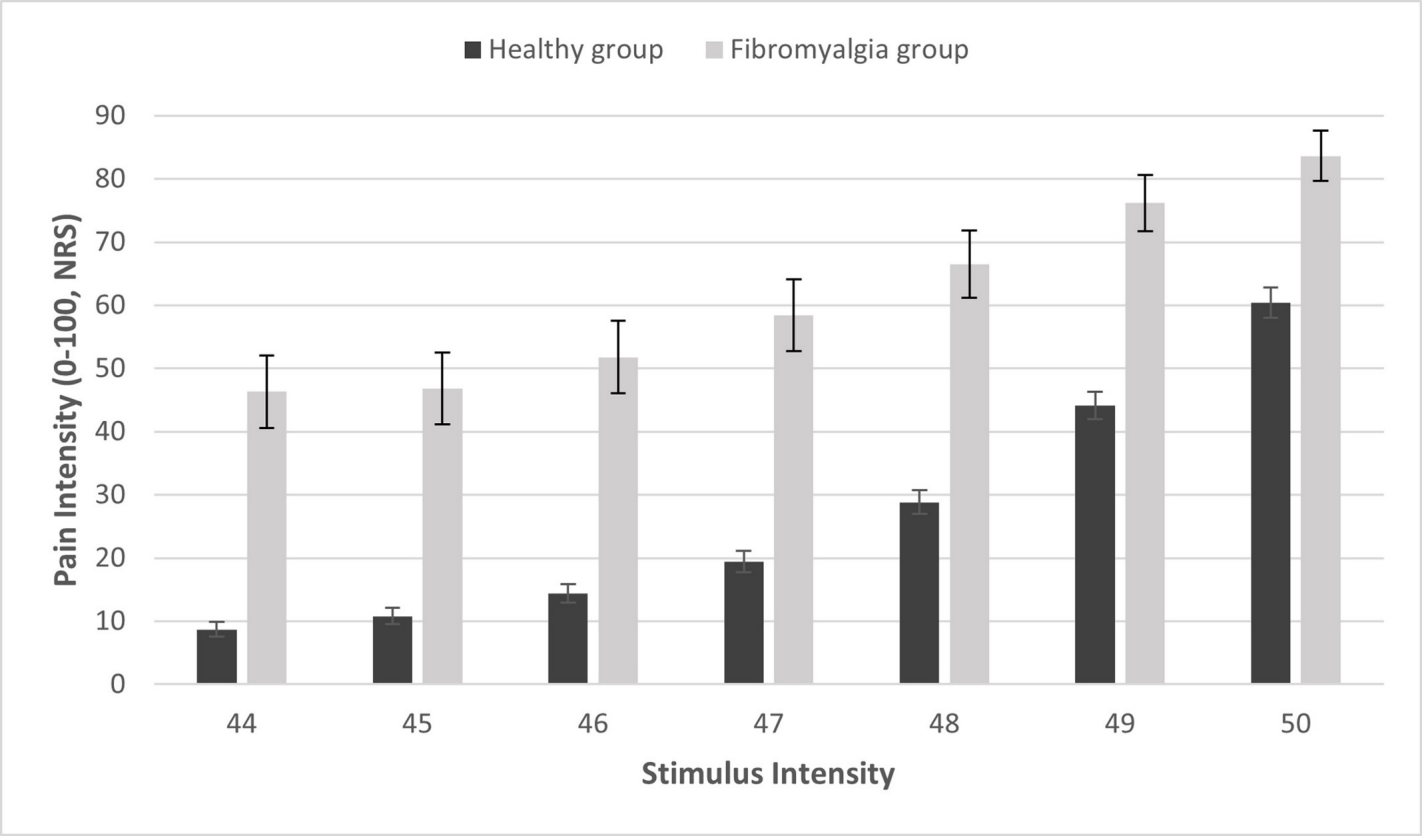
Mean pain scores in response to the 7 FAST stimulus intensities. Black and grey bars represent the average pain scores in response to the 7 repetitions of the stimulus at each intensity, for healthy and Fibromyalgia patients, respectively. Error bars represent the standard error of the mean.

The FAST procedure involves a total of 49 stimuli: 7 applications of 7 different temperature intensities (44, 45, 46, 47, 48, 49, and 50°C). Mean pain intensity ratings for each of these temperatures are depicted in Fig 1. In the healthy subjects’ cohort, Group means ± SD pain responses ranged from 8.70 ± 12.59 for the lowest stimulus (44°C) to 60.40 ± 26.06 for the highest (50°C). In the Fibromyalgia cohort pain responses ranged from 46.32 ± 30.68 for the lowest stimulus (44°C) to 83.65 ± 21.61 for the highest (50°C).

Further analysis indicates that in both cohorts, significant differences were found between pain responses to each temperature (Friedman’s tests, chi-square = 623.89; *P* < 0.001 for the healthy subjects and chi-square = 125.79; *P* < 0.001 for the Fibromyalgia). In the healthy subjects’ cohort, post hoc Wilcoxon test revealed significant differences between all stimuli intensities (largest p-value of 0.004). In the Fibromyalgia cohort, post hoc Wilcoxon test revealed significant differences between each 2 stimuli intensities (*P* < 0.05), except between 44°C and 45°C (*P* = 0.510).

Regarding of differences in sensitivity to pain between the cohorts, Mann-Whitney test revealed significant between-group differences in response to all stimuli intensities (*P*<0.001 for all, see Fig 1). As such, the mean pain scores for the lowest stimulus (44°C) in the Fibromyalgia cohort, the mean ± SD pain was 46.32 ± 30.68, while in the healthy cohort it was only 8.70 ± 12.59 (U = 406.500, p<0.001). For the highest stimulus temperature (50°C), the Fibromyalgia cohort had a group mean ± SD of 83.65 ± 21.61, while for the healthy 60.40 ± 26.06 (U = 826.500, p<0.001).

Descriptive statistics of the 2 FAST outcomes (*R*^2^ and ICC) are presented in Table 3. The means of the within-subject variability measures significantly (p<0.001) differ between the cohorts, with larger variability (lower values of *R*^2^ and ICC) found in the Fibromyalgia group, compared to controls.

**Table 3:** FAST outcome measures and mean digit span scores.

		HC	FM	*p-value*
**FAST**				
	***R*** ^**2**^			0.001
	Mean±SD	0.559±0.14	0.469±0.15	
	Median	0.590	0.493	
	Range	0.0180–0.762	0.006–0.701	
	**ICC**			<0.001
	Mean±SD	0.669±0.19	0.546±0.19	
	Median	0.711	0.583	
	Range	0.073–0.893	−0.139–0.774	
**Digit Span**				
	**Forward**			0.002
	Mean±SD	9.58±1.87	8.14±2.17	
	Median	10.00	8.00	
	Range	6.00–15.00	4.00–12.00	
	**Backward**			0.914
	Mean±SD	5.30±2.06	5.24±1.84	
	Median	6.00	6.00	
	Range	1.00–10.00	2.00–8.00	
	**Total**			0.046
	Mean±SD	14.88±3.19	13.38±3.31	
	Median	14.00	14.00	
	Range	7.00–25.00	8.00–20.00	

Note: HC = healthy cohort; FM = fibromyalgia group; FAST = focused analgesia selection test; SD = standard deviation; ICC = intraclass correlation coefficient.

### Assessment of short-term memory via the digit span

Table 3 also present summary of the digit span test results. Healthy participants had a mean score ± SD of 9.58 ± 1.87 for the forward-order test and 5.29 ± 2.05 for the reverse-order test. The mean digit span total score was 14.86 ± 3.18. Fibromyalgia patients had significant lower scores: 8.14 ± 2.17 for the forward-order (p = 0.002) test and for the total score of 13.38 ± 3.31 (p = 0.046), while no significant differences were found in the reverse-order test (p = 0.914).

### Correlations between within-subject variability of pain intensity reports and short-term memory

**Fig 2:**
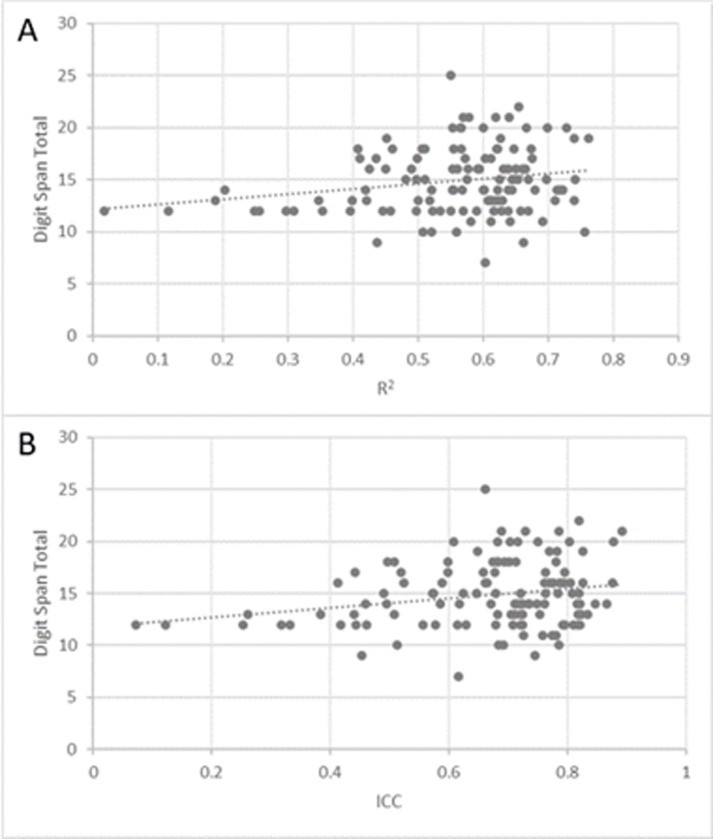
Correlation between mean digit span total scores and FAST R^2^ and ICC in the healthy volunteers’ cohort. Correlations between the Digit span total scores and the FAST outcomes in healthy volunteers. Fig 2A and 2B present the correlations between Digit span total and the FAST R^2^ and FAST ICC, respectively.

In the healthy volunteers’ cohort, a trend toward positive correlations were found between the digit span total scores and the FAST *R*^2^ (Spearman’s *r* = 0.174, *P* = 0.056) and the FAST ICC (Spearman’s r = 0.152, *P* = 0.096) (Fig 2A and 2B). No significant correlations were found between the two FAST outcome and the digit span forward or backward subscales in this group.

**Fig 3:**
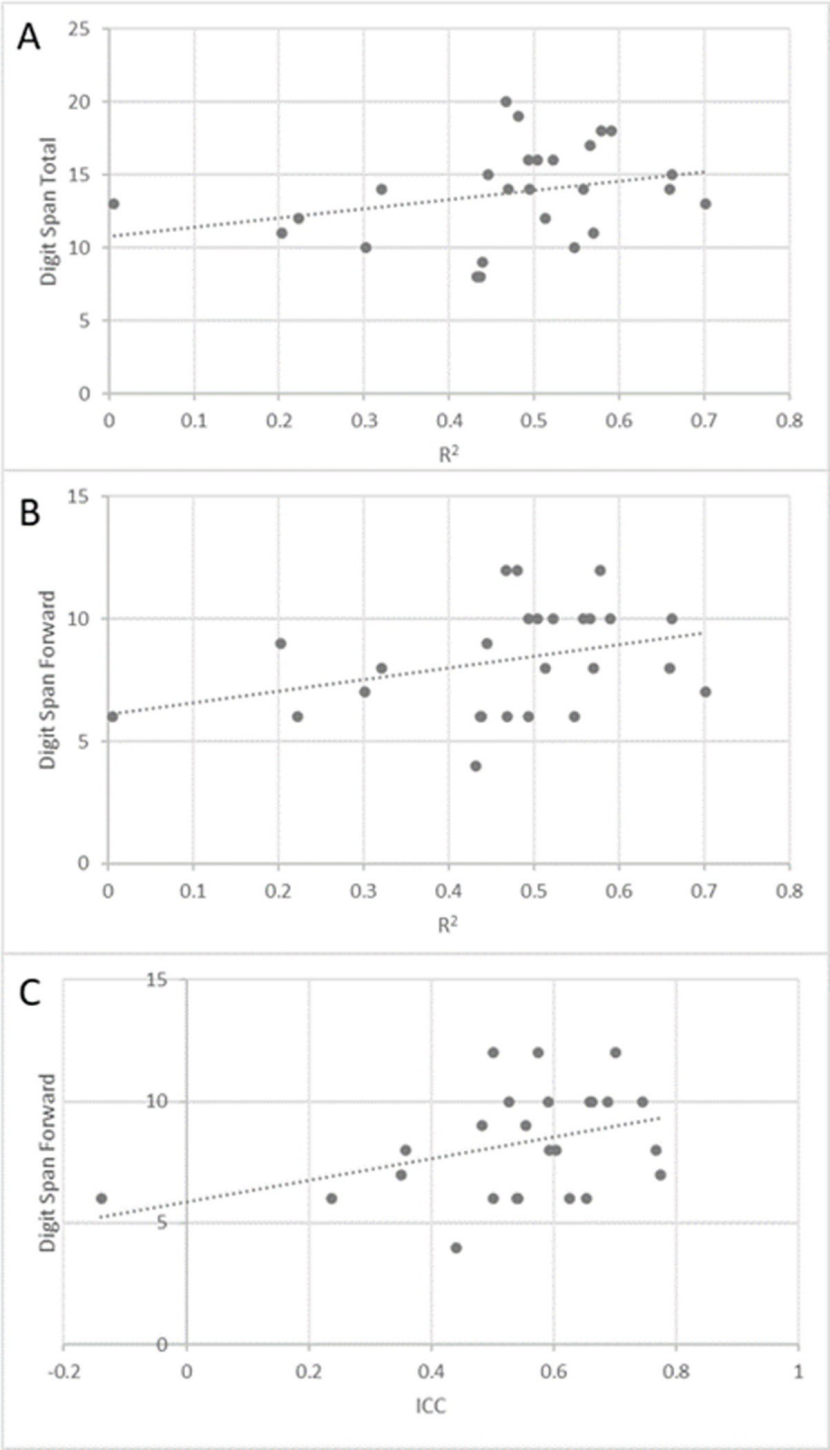
Correlations between forward-order and total digit span scores and FAST R^2^ and ICC, Fibromyalgia group. Correlations between the Digit span total and forward scores and the FAST outcomes in the fibromyalgia group. Fig 3A shows the correlation between the Digit span total and the FAST R^2^. Fig 3B and 3C present the correlations between Digit span forward and the FAST R^2^ and FAST ICC, respectively.

In the Fibromyalgia group, significant positive correlations were found between digit span total score and FAST *R*^2^ (Spearman’s *r* = 0.394, *P* = 0.046, Fig 3A) and between the forward-order digit span scores and the FAST *R*^2^ and ICC (Spearman’s *r* = 0.418, *P* = 0.034; Spearman’s *r* = 0.399, *P* = 0.043, Fig 3B and 3C, respectively).

Notably, no significant correlations were found between the sensitivity to pain (mean of pain intensity reports of all stimuli) and any of the digit span outcomes, in both cohorts.

## Discussion

This report summarizes the results of two studies in which the relationships between short-term memory and the within-subject variability of pain reports were investigated in 2 populations: healthy participants and participants with Fibromyalgia.

Our main finding was that regardless of the differences between the two cohorts, short-term memory was found to be correlated (significantly, in the patient’s cohort, and with a statistical trend in the healthy controls) with the within-subject variability. Our interpretation for this finding is that poor short-term memory may be contributing to the within-subjects’ variability captured by the FAST procedure. Similar to the variability in any other measure, the variability of pain intensity reported during the FAST procedure is derived by two components: true variability due to changes in perception, and error variability due to any measurement error [39, 40]. Hence, poorer ability to remember recent events could contribute to the error variability component of pain intensity reports. In other words, to demonstrate low variability in the FAST procedure, the participant needs to undertake a cognitive task during which the present pain sensation is mentally compared to the sensations that were experienced recently (in response to previous stimuli). Good short-term memory supports this mental process of comparing the intensity of current pain to pain experienced very recently, hence contributing to low variability in the FAST results. Furthermore, the lack of correlations between pain sensitivity and short-term memory further suggest that the latter interfere with the mental process of pain evaluation, rather than affecting pain perception per-se. A possibly related finding is derived from electroencephalogram studies, in which larger neuronal noise, known to be associated with poor short-term memory [41] was found in Fibromyalgia patients [42].

The relationships between pain and cognition has long been acknowledged and has mainly focused on complaints of the reduced cognitive abilities of patients with chronic pain. Both clinical and preclinical studies provide further evidence of these changes in cognitive abilities in acute and chronic pain [43, 44]. Chronic pain patients demonstrate lower cognitive performance than do controls in many different cognitive domains such as attention [45], decision-making [46], cognitive flexibility [47], processing speed [48], and both short- and long-term memory [49–53]. Three literature reviews [54–56] found a consistent moderate yet significant decline of short-term memory in patients with chronic pain.

In addition to the diminishing effect of clinical pain on cognitive abilities, including short-term memory, evidence also suggests that experimental pain has similar effects on memory. A few studies show that when healthy subjects experience acute induced experimental pain, their short-term memory is impaired when it is assessed concurrently but it is not impaired when tested after the painful experience [57–60]. This finding suggests that the effects of experimentally induced pain on memory and perhaps on other cognitive abilities is limited, mainly due to cognitive load. Whether the research addresses acute or chronic pain, the entire literature is focused on the negative effects of pain on cognitive abilities (including memory). To our best knowledge, our findings are the first to focus on the contribution of memory to pain evaluation.

The International Association for the Study of Pain’s recently revised definition of pain, which now includes the term “resembling” to ensure that in patients with limited cognitive resources, such as infants or older individuals with dementia, pain could still exist [12, 61]. Our findings are in line with this revised definition. However, instead of focusing on extreme developmental phases or clinical situations, we focus on normal cognitive differences, which could also have an impact on pain processing, assessment, and reports. This interpretation is in line with the difference in the strength of correlations found in the two cohorts in our study. While in the Fibromyalgia strong significant correlations were found, in the healthy cohort only weaker trend of correlations were seen. It might be that these associations are clearer in chronic pain populations, in which memory ability might be compromised [53]. Future research is needed to identify if rehabilitative approaches, on top of their effects on pain, would positively affect memory or other cognitive functions [62–65].

Short-term memory is generally conceptualized as a type of temporary storage of limited capacity, in contrast with long-term memory, which can store (theoretically) unlimited amounts of information during a person’s lifetime [66, 67]. The digit span task includes two subscales, the forward and backward tests, both of which assess recall of verbal information, with one difference: In the forward-order task, subjects are asked to repeat a series of numbers verbally presented. This task requires retention and reproduction of information. In the backward -order task, participants must repeat the numbers in their reversed order, a task that requires additional mental manipulation of the information [66, 67]. It is generally accepted that the forward-order task assesses short-term memory and is a passive function, whereas the reverse-order task, which assesses working memory, is considered a higher-level executive function [68]. In both cohorts, correlations were seen between the forward and total score, but not with backward task, suggesting that it is the short-term memory (rather than the working memory) that is related to the variability expressed in the FAST.

The significant between-group differences found in short-term memory assessments are not unexpected, except for the participants’ performance on the backward -order task, which did not differ between the groups. Given that the two cohorts differ in various characteristics, we cannot attribute these differences (or lack thereof) in short-term memory to a specific cause.

Three main limitations of the current study deserve discussion: (1) The Fibromyalgia patients were found to be more sensitive to pain and to express greater variability of their pain intensity scores than controls. While these differences could be attributed to the characteristics of the cohorts (age, sex, and the difference in the number of participants in each group), we cannot ignore that the two cohorts were investigated in two different laboratories, located in different countries, which could contribute to these differences. (2) The assessment of short-term memory might be sensitive to the modality being used [69, 70], hence, it might be that short-term memory as assessed by the Digit span test is not the most relevant construct to investigate with relations with pain assessment, which relay on a different sensory modality. Future studies should investigate possible associations between short-term memory assessed via other modalities, and the within-subjects variability of pain intensity reports. (3) It cannot be excluded that differences in within-subject variability in pain reports could be related to other clinical or individual differences between the two cohorts.

In summary, associations were found between short-term memory and the within-subject variability of pain reports captured in the FAST procedure. While the associations were significant in the Fibromyalgia cohort, they were only marginal in the healthy volunteers’ cohort, suggesting that these relations are more relevant in clinical population. The most reasonable interpretation of these results is that short-term memory might be essential to the cognitive process of pain evaluation in this, and perhaps other experimental tasks. Future studies should investigate the possible relationship between clinical day-to-day within-subject variability, as captured by pain diaries, which are the clinical equivalent of the variability captured in the FAST, and memory abilities, which could be assessed via various tests [70, 71]. If there is a relationship, it will further confirm our hypothesis that at least some of the within-subject variability of pain is due to error measurement.

## Supporting information

S1 File (XLSX)
